# Plastome phylogenomic analysis reveals evolutionary divergences of Polypodiales suborder Dennstaedtiineae

**DOI:** 10.1186/s12870-022-03886-1

**Published:** 2022-11-02

**Authors:** Jin-Mei Lu, Xin-Yu Du, Li-Yaung Kuo, Atsushi Ebihara, Leon R. Perrie, Zheng-Yu Zuo, Hui Shang, Yi-Han Chang, De-Zhu Li

**Affiliations:** 1 Germplasm Bank of Wild Species, Kunming Institute of Botany, Chinese Academy of Sciences, 132 Lanhei Road, Kunming, 650201 China; 2 Institute of Molecular and Cellular Biology, National Tsing Hua University, Hsinchu, 30013 Taiwan; 3 Department of Botany, National Museum of Nature and Science, 4–1–1 Amakubo, Tsukuba, Ibaraki 305–0005 Japan; 4 Museum of New Zealand Te Papa Tongarewa, Cable Street, Wellington, 6011 New Zealand; 5 Shanghai Chenshan Plant Science Research Center, Chinese Academy of Sciences, Shanghai Chenshan Botanical Garden, Shanghai, 201602 China; 6 Taiwan Forestry Research Institute, Taipei, 10066 Taiwan

**Keywords:** Dennstaedtiaceae, Plastid phylogenomics, Monachosoraceae, *Monachosorum*, Monophyly, Morphological character, Divergence time

## Abstract

**Background:**

Polypodiales suborder Dennstaedtiineae contain a single family Dennstaedtiaceae, eleven genera, and about 270 species, and include some groups that were previously placed in Dennstaedtiaceae, Hypolepidaceae, Monachosoraceae, and Pteridaceae. The classification and phylogenetic relationships among these eleven genera have been poorly understood. To explore the deep relationships within suborder Dennstaedtiineae and estimate the early diversification of this morphologically heterogeneous group, we analyzed complete plastomes of 57 samples representing all eleven genera of suborder Dennstaedtiineae using maximum likelihood and Bayesian inference.

**Results:**

The phylogenetic relationships of all the lineages in the bracken fern family Dennstaedtiaceae were well resolved with strong support values. All six genera of Hypolepidoideae were recovered as forming a monophyletic group with full support, and *Pteridium* was fully supported as sister to all the other genera in Hypolepidoideae. Dennstaedtioideae (*Dennstaedtia s.l.*) fell into four clades with full support: the *Microlepia* clade, the northern *Dennstaedtia* clade, the *Dennstaedtia globulifera* clade, and the *Dennstaedtia s.s.* clade. *Monachosorum* was strongly resolved as sister to all the remaining genera of suborder Dennstaedtiineae. Based on the well resolved relationships among genera, the divergence between *Monachosorum* and other groups of suborder Dennstaedtiineae was estimated to have occurred in the Early Cretaceous, and all extant genera (and clades) in Dennstaedtiineae, were inferred to have diversified since the Late Oligocene.

**Conclusion:**

This study supports reinstating a previously published family Monachosoraceae as a segregate from Dennstaedtiaceae, based on unique morphological evidence, the shady habitat, and the deep evolutionary divergence from its closest relatives.

**Supplementary Information:**

The online version contains supplementary material available at 10.1186/s12870-022-03886-1.

## Background

The purpose of a phylogeny is the construction of a genealogical tree of organisms, its substantiation, and interpretation [1]. Classification can provide a framework to identify plants and to conduct evolutionary and physiological studies, and it can serve us better if we can name those groups that are readily recognizable and characterized by morphological diagnosability and homogeneity, at least at higher ranks (families and orders) [2]. A comprehensive phylogeny‐based classification of pteridophytes (including ferns and lycophytes) was established by the Pteridophyte Phylogeny Group in 2016 and has been widely accepted and cited in recent years [3]. Given the limitations of sampling and research methods, the PPG classification should not be the final decision on lycophytes and ferns classification, although it incorporated all phylogenetic data available at the time.

Numerous phylogenetic studies have been conducted since the publication of the PPG classification [3], especially on those globally distributed families, e.g., Lycopodiaceae [4], Polypodiaceae [5, 6], Pteridaceae [7], and Thelypteridaceae [8, 9]. The increase in the number of samples, especially in regions and groups with low sampling density, and the accumulation of molecular data, have improved the ability of taxon delimitation and identification and prompted the publication of some new taxa. One new family was published by Zhou et al. [10], and some new genera have continued to be recognized (e.g., [11, 12, 13, 14, 15, 16]). The systematic positions of several remaining enigmatic groups in the PPG classification [3] were resolved [5, 6, 17, 18, 19, 20]. In addition, some hybrid genera have been found (e.g., × *Cyclobotrya*, [21]; × *Woodsimatium* Li Bing Zhang, N.T. Lu & X.F. Gao, [22]; reviewed in [23]).

Polypodiales were first recognized as a distinct taxonomic group at the beginning of the nineteenth century, and its monophyly has been strongly supported by a unique morphological character (the interruption of the vertical annulus by a sporangium stalk) and molecular studies (e.g. [17, 18, 24, 25, 26, 27],). During the nineteenth century nearly all leptosporangiate ferns were placed in Polypodiaceae in the broadest sense (Polypodiaceae *s.l.*). Ching [28] was the first to recognize the heterogeneity of Polypodiaceae *s.l.*, and divided it into 33 families. Smith et al. [2] recognized 15 families in Polypodiales, while Christenhusz et al. [29] included two newly described families and recognized 23 families in Polypodiales. In the following five years, four new families, Hemidictyaceae, Arthropteridaceae, Didymochlaenaceae, and Desmophlebiaceae, were described [30, 31, 32, 33]. PPG I [3] accepted Hemidictyaceae, Didymochlaenaceae, and Desmophlebiaceae, but not Arthropteridaceae, and recognized 26 families in six suborders for Polypodiales. Zhou et al. [10] established a new family of Pteridryaceae (comprising four genera) as a segregate from Tectariaceae based on the criteria of monophyly and the diagnosability, and they also advocated for the recognition of Arthropteridaceae. Hence ten new families have been published and/ or recognized in Polypodiales, specifically in suborder Aspleniineae (5 families), suborder Polypodiineae (3 families), suborder Lindsaeineae (1 family), and suborder Saccolomatineae (1 family) in the last two decades. But the remaining two suborders Pteridineae and Dennstaedtiineae each contain one relatively heterogeneous family.

Polypodiales suborder Dennstaedtiineae were recently considered to contain one family Dennstaedtiaceae, eleven genera and about 270 species [2, 3, 34, 35, 36], and include some groups that were previously placed in Hypolepidaceae (e.g., *Hypolepis* Bernh.), Monachosoraceae (*Monachosorum* Kunze), and Pteridiaceae (*Pteridium* Gled. ex Scop.) (Table 1). Ching [28] included *Dennstaedtia* Bernh., *Leptolepia* Prantl, *Microlepia* C. Presl, *Oenotrichia* Copel., and four additional genera of tribe Saccolomeae in his Dennstaedtiaceae, and *Hypolepis* in Hypolepidaceae, and he placed *Monachosorum* and *Ptilopteris* Hance in Monachosoraceae. Pichi Sermolli [37] classified *Monachosorum* and *Ptilopteris* of Monachosoraceae Ching into Dennstaedtiaceae, and circumscribed about ten genera in Dennstaedtiaceae, while he assigned *Blotiella* R.M. Tryon*, Histiopteris* (J. Agardh) J. Sm., *Hypolepis*, *Lonchitis* L.*, Paesia* A. St.-Hil.*,* and *Pteridium* in Hypolepidaceae. Kramer [38] circumscribed Dennstaedtiaceae more broadly and included subfamily Saccolomatoideae (1 genus), subfamily Dennstaedtioideae (10 genera), and subfamily Lindsaeoideae (5 genera). Early molecular studies showed that Monachosoraceae were nested within Dennstaedtiaceae while the lindsaeoid ferns were relatively distant from Dennstaedtiaceae [26, 39]. Further expanded phylogenetic analyses of Dennstaedtiaceae [40] showed that *Coptodipteris* Nakai & Momose; *Leptolepia*, *Microlepia*, and *Oenotrichia* fell within *Dennstaedtia*. Shang et al. [35] described a new genus *Hiya* H. Shang from *Hypolepis* based on combined evidence of morphology, cytology, and molecular phylogeny. The molecular phylogeny based on five plastid DNA markers further recovered three subfamilies in Dennstaedtiaceae: Dennstaedtioideae, Hypolepidoideae, and Monachosoroideae [34], which is consistent with the study of the morphological anatomy of the rhizomes [41]. Most genera (except for *Dennstaedtia*) in Dennstaedtiaceae have been supported as monophyletic; However, the phylogenetic relationships among these genera have not been resolved [34, 35, 40, 41].

**Table 1:** Comparison of family and genera concepts in taxonomy and phylogeny-based classifications

Genus	PPG I (2016) [3]	Smith et al. (2006) [2]	Kramer (1990a, b) [38, 42]	Pichi Sermolli (1970) [37]	Ching (1940) [43]
*Blotiella* R.M.Tryon (1962)	Dennstaedtiaceae	Dennstaedtiaceae	Dennstaedtiaceae subfamily Dennstaedtioideae	Hypolepidaceae	
*Dennstaedtia* Bernh	Dennstaedtiaceae	Dennstaedtiaceae	Dennstaedtiaceae subfamily Dennstaedtioideae	Dennstaedtiaceae	Dennstaedtiaceae
*Histiopteris* (J.Agardh) J.Sm	Dennstaedtiaceae	Dennstaedtiaceae	Dennstaedtiaceae subfamily Dennstaedtioideae	Hypolepidaceae	Pteridiaceae
*Hypolepis* Bernh	Dennstaedtiaceae	Dennstaedtiaceae	Dennstaedtiaceae subfamily Dennstaedtioideae	Hypolepidaceae	Hypolepidaceae
*Leptolepia* Mett. ex Prantl	Dennstaedtiaceae	Dennstaedtiaceae	Dennstaedtiaceae subfamily Dennstaedtioideae	Dennstaedtiaceae	Dennstaedtiaceae
*Microlepia* C. Presl	Dennstaedtiaceae	Dennstaedtiaceae	Dennstaedtiaceae subfamily Dennstaedtioideae	Dennstaedtiaceae	Dennstaedtiaceae
*Oenotrichia* Copel	Dennstaedtiaceae	Dennstaedtiaceae	Dennstaedtiaceae subfamily Dennstaedtioideae	Dennstaedtiaceae	Dennstaedtiaceae
*Paesia* St. Hilaire	Dennstaedtiaceae	Dennstaedtiaceae	Dennstaedtiaceae subfamily Dennstaedtioideae	Hypolepidaceae	Pteridiaceae
*Pteridium* Gled. ex Scop	Dennstaedtiaceae	Dennstaedtiaceae	Dennstaedtiaceae subfamily Dennstaedtioideae	Hypolepidaceae	Pteridiaceae
*Lonchitis* L	Lonchitidaceae	Lindsaeaceae	Dennstaedtiaceae subfamily Dennstaedtioideae	Hypolepidaceae	Pteridiaceae
*Monachosorum* Kunze	Dennstaedtiaceae	Dennstaedtiaceae	Monachosoraceae	Dennstaedtiaceae	Monachosoraceae
*Ptilopteris* Hance	= *Monachosorum*		= *Monachosorum*	Dennstaedtiaceae	Monachosoraceae
*Hiya* H. Shang (2018) [35]					
*Coptidipteris* Nakai & Momose (1937)	= *Dennstaedtia*	Dennstaedtiaceae			
*Saccoloma* Kaulfuss			Dennstaedtiaceae subfamily Saccolomatoideae	Dennstaedtiaceae	Dennstaedtiaceae
*Ormoloma* Maxon			= *Saccoloma*	Dennstaedtiaceae	Dennstaedtiaceae
*Ithycaulon* Copel			= *Saccoloma*	Dennstaedtiaceae	Dennstaedtiaceae
*Orthiopteris* Copel	=* Saccoloma*			Dennstaedtiaceae	Dennstaedtiaceae

In this study, we used a phylogenomic approach to resolve the phylogenetic relationships among all genera in Polypodiales suborder Dennstaedtiineae (i.e., Dennstaedtiaceae *s.l.*, Dennstaedtiaceae sensu PPG I), and test the phylogenetic and taxonomic positions of *Monachosorum*. Special attention was paid to explore the deep divergences in suborder Dennstaedtiineae using the analyses of morphological characters and the estimation of the divergence time.

## Results

### Characteristics of plastomes and datasets

Forty-four complete or nearly complete plastomic sequences of Polypodiales suborder Dennstaedtiineae were generated in this study (Additional file 1). All sequenced plastomes showed a high similarity in genome structure and gene content to the published Dennstaedtiaceae plastomes. The GC content of novel plastomes ranged from 41.5% (*Pteridium caudatum*) to 45.5% (*Monachosorum maximowiczii*), and the size of the plastomes ranged from 147,263 bp (*Leptolepia novae-zelandiae*) to 158,547 bp (*Histiopteris herbacea*) (Additional file 1). *Monachosorum* showed the highest sequence divergence between genera in suborder Dennstaedtiineae.

### Phylogenetic relationships within Dennstaedtiaceae s.l.

**Fig. 1:**
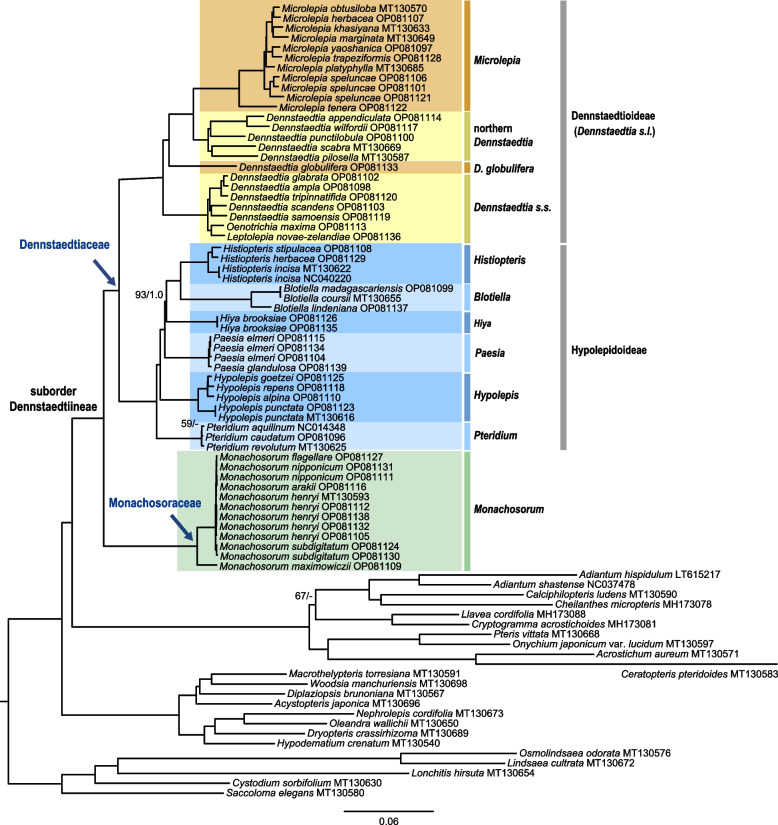
Phylogram of Polypodiales suborder Dennstaedtiineae based on complete chloroplast genome sequences. Tree topology with branch length indicated was based on maximum likelihood (ML) analysis using the GTR + H4 model. The names of major nodes are indicated. Support values indicated were from ML analyses using the GTR + H4, and Bayesian inference (BI) analysis using the GTR + I + G model. Support values, including bootstrap support values (BS) and Bayesian confidence values (PP), are indicated along the branches unless all BS and PP are 100% or 1.0

All four analyses (Table 2) recovered identical three major lineages previously recognized as subfamilies of Dennstaedtiaceae: Dennstaedtioideae, Hypolepidoideae, and Monachosoroideae of the currently defined Dennstaedtiaceae (Fig. 1). The topologies from different analyses were almost identical, only with minor differences in *Pteridium* (Fig. 1; Additional files 2 and 3). All relationships among subfamilies, genera, and clades were fully or strongly supported (MLBS > 90%, BIPP = 1.0).

**Table 2:** Data characteristics with models selected and used in different analyses

Data set	No. of taxa (ingroup)	No. of sites (bp)	Variable/Parsimony informative sites (%)	Methods	Models
Pt	80 (57)	124,738	77,883 (62.4%)/63,703 (51.1%)	ML	GTR + H4
				BI	GTR + I + G
CDS	80 (57)	72,828	42,547 (58.4%)/35,797 (49.2%)	ML	PartitionFinder2 defined
				BI	GTR + I + G

All subfamilies and genera were recovered as monophyletic with full support, except for *Dennstaedtia* (Fig. 1). *Monachosorum* was resolved as sister to all the remaining genera. Within the Hypolepidoideae, *Pteridium* was fully supported as a sister of all the other genera, followed sequentially by *Hypolepis*, *Paesia*, *Hiya*, *Blotiella*, and *Histiopteris*. The *Dennstaedtia s.l.* clade (i.e., Dennstaedtioideae sensu Schwartsburd et al.) contained four clades—the *Microlepia* clade, the northern *Dennstaedtia* clade, the *D. globulifera* clade, and the *Dennstaedtia s.s.* clade. *Leptolepia* and *Oenotrichia* were nested within the *Dennstaedtia s.s.* group.

### Divergence time estimation

**Fig. 2:**
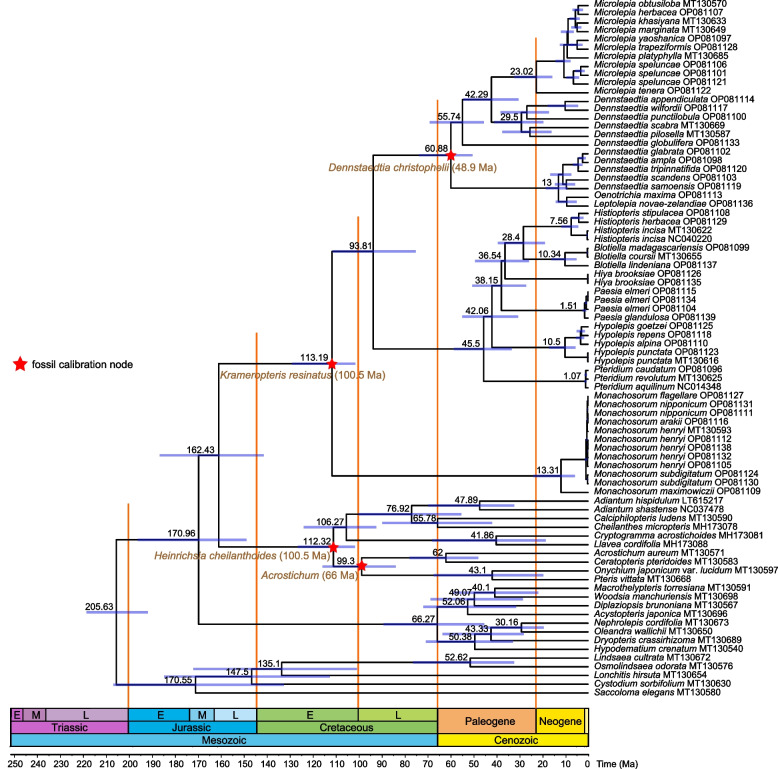
Chronogram of Polypodiales suborder Dennstaedtiineae. Blue horizontal bars on the nodes indicate 95% credible intervals of the divergence time estimates (95% HPD), and numbers on the bars indicate the median age (Ma). Geological timescale and subdivisions: E, Early; L, Late; M, Middle

Based on the dating results from the BEAST analysis using 191.62–219.87 Ma as the minimum–maximum secondary age constraint of the root (the stem of Polypodiales) combined with four fossil records, the currently defined Dennstaedtiaceae were estimated to have originated at 162.43 Ma (95% HPD: 141.29, 186.63 Ma), with its crown group dated to 113.19 (101.42, 129.04) Ma (Fig. 2). The early diversification of Dennstaedtiaceae mainly occurred during the Early Cretaceous period, giving rise to Dennstaedtioideae and Hypolepidoideae (93.81 (75.04, 111.23) Ma), and Monachosoroideae.

The second diversification occurred mainly during the Paleogene, resulting in the radiation of Dennstaedtioideae and Hypolepidoideae. The crown ages of Dennstaedtioideae and Hypolepidoideae were estimated to be 60.88 (50.31, 73.6) Ma and 45.5 (33.24, 58.41) Ma, respectively. All genera (and clades) in Dennstaedtiaceae were inferred to have started to diversify in the Oligocene or later (29.5–1.07 Ma), and the diversification of extant *Monachosorum* was inferred to have begun only at about 13.31 (5.68, 23.81) Ma.

## Discussion

### The phylogenetic position of suborder Dennstaedtiineae in Polypodiales

In his phylogenetic classification of the homosporous ferns, Nayar [44] proposed that Dennstaedtiaceae had a close relationship with Dicksonioideae and Thyrsopteridoideae, and the family gave rise to Lindsaeaceae and Hypolepidaceae in order Cyatheales. Molecular phylogenetic studies suggested that Dennstaedtiaceae were sister to a clade comprising Pteridiaceae and the eupolypods [26, 27, 45], or sister to the eupolypods [25, 46, 47, 48], or sister to Pteridiaceae [17, 18].

Our phylogeny based on 57 plastomes from Polypodiales suborder Dennstaedtiineae and 23 plastomes from other families in Polypodiales supports the monophyly of the currently defined suborder Dennstaedtiineae as in the PPG classification. All four analyses based on the plastomic and CDS data sets resolve suborder Dennstaedtiineae to be sister to suborder Pteridineae with full support values in the present study. A recent study showed that Dennstaedtiineae (Dennstaedtiaceae) share a 3-codon deletion in the *ndh*B gene with Pteridineae (Pteridiaceae) [17]. There is morphological evidence supporting the sister relationship between Dennstaedtiaceae and Pteridiaceae, rather than that supporting the sister relationship between Dennstaedtiaceae and the eupolypods. The marginal (false) sori protected by reflexed indusia formed by reflexed upper side of the margins of pinnae or leaflets are found in some genera of Dennstaedtiaceae (*Paesia* and *Pteridium*) and most genera of Pteridaceae (e.g., [49, 50]), but never found in members of the eupolypod clade. Secondly, a solenostele can be found in both Dennstaedtiaceae and Pteridaceae, but never in eupolypods. Furthermore, eupolypod families are characterized by having a circumendodermal band (CB, a distinctive, second, innermost layer of the fundamental tissue adjacent to the innermost endodermis) that surrounds the leaf traces in the petioles [51], while there is no CB in the early-diverging members of Polypodiales (e.g., Dennstaedtiaceae, Lindsaeaceae, Pteridaceae, and Saccolomataceae).

### Plastome phylogeny of Dennstaedtiaceae

Previous research on morphology, development, and ultrastructure was considered to provide additional phylogenetic insights [39]. There are many different morphological characteristics in sporophytes among the genera of Dennstaedtiaceae, making the systematic circumscription of the family difficult.

The previous molecular phylogenetic studies showed that Dennstaedtiaceae comprises three clades (subfamilies); however, the relationships among the three clades were not well supported [34]. The relationships among Dennstaedtioideae and Hypolepidoideae, genera, and clades are strongly supported with full support values in our analyses. *Monachosorum* is strongly supported as sister to all the remaining genera in Dennstaedtiaceae. All six genera of Hypolepidoideae [34] are recovered as forming a monophyletic group with full support. *Pteridium* is fully supported as sister to the remaining Hypolepidoideae, which is consistent with most previous studies [35, 40, 52] and the morphological characteristics of spores. *Hiya* is strongly supported as sister to the assembly of *Blotiella* and *Histiopteris* by 93% ML bootstrap support values and 1.0 Bayesian confidence values (Fig. 1). The present result differs from previous studies [34, 35], in which *Paesia* was sister of the *Blotiella*-*Histiopteris* clade although this topology was only weakly supported.

Species of the *Dennstaedtia s.l.* clade are distributed mainly in the tropics and subtropics and grow in open habitats. They are edge-colonizers, and have long-creeping rhizomes, marginal or submarginal sori protected by cup-shaped or half-cup-shaped indusia, and trilete spores. Our study supports a broadly defined *Dennstaedtia s.l.* and does not corroborate the monophyly of *Dennstaedtia s.s.*, which is consistent with three previous studies based on a single or a few chloroplast markers [34, 35, 40]. Our study suggests that *Dennstaedtia s.l.* falls into four clades each with full support, and the newly identified *D. globulifera* clade is the sister group of the *Microlepia* clade and the northern *Dennstaedtia* clade. Morpho-anatomical studies of the rhizomes of Dennstaedtiaceae [41] showed only the outermost layer of the pith is occupied by sclerified parenchyma in *Dennstaedtia globulifera* (Poir.) Hieron., while in species of the northern *Dennstaedtia* clade sclerified parenchyma occupies almost the entire pith. More recently, a phylogenetic study of Dennstaedtioideae based on four chloroplast markers also confirmed that Dennstaedtioideae consist of four clades, and proposed a classification of four genera corresponding to the four clades in Dennstaedtioideae [53]. The type species of *Leptolepia* and *Oenotrichia* were sampled in our study; however, the samples of the type species of *Microlepia* (*M. polypodioides* (Sw.) C. Presl) and *Dennstaedtia* (*Dennstaedtia flaccida* (G. Forst.) Bernh) were not sampled in the present study. When the *rbc*L sequence of *D. flaccida* (GenBank accession: MT657694) was compared with those of our samples, *D. flaccida* is nested within the *Microlepia* clade and was closely related to *M. speluncae* (L.) T. Moore (not shown). Another study based on four chloroplast markers also proposed inclusion of *D. flaccida* in *Microlepia* [53]. We suggest that the specimens of the type species *M. polypodioides* need to be further sampled to test the circumscription of this genus. Until then, we prefer the retention of a more inclusive *Dennstaedtia*.

### On the isolated position of Monachosorum in suborder Dennstaedtiineae

*Monachosorum* Kunze is a small genus in Asia comprising approximately six species, mainly distributed in the temperate regions. Morphologically, *Monachosorum* is somewhat related to the davallioid (in the relationship of the lamina of a leaflet to the rachis bearing it), thelypteroid ferns (in petiolar structure and vascular cylinder), dennstaedtioid ferns (in frond form, the absence of scales, and the absence of hair on the gametophytes), and anogrammoid ferns (in the forked and open veins, exindusiate sori, and trilete spores with perine).

Ching [28] recognized Monachosoraceae for the first time and included two Asian genera *Monachosorum* and *Ptilopteris* in it, and he proposed that Monachosoraceae was related to the thelypterioid and the athyrioid ferns. However, Ching [28] did not provide a Latin description and thus failed to validly publish this family in 1940. Ching [43] validated the family Monachosoraceae in his classification of Chinese fern families and genera. Some pteridologists [42, 54] treated *Monachosorum* as the type genus of the Monachosoraceae, and some others did not recognize the family and considered that *Monachosorum* had a close relationship with Dennstaedtiaceae [37, 44, 55, 56, 57, 58], Thelypteridaceae [59], Davalliaceae [60], Cyatheoids [61], or the aspidioid ferns [62]. The early molecular phylogenetic study by Wolf [39] implied including *Monachosorum* in Dennstaedtiaceae although there were low bootstrap value and the ambiguity in the phylogenetic tree.

There is a curious mixture of primitive and advanced characters in *Monachosorum* [54]. The most distinctive feature of *Monachosorum* is the production of the typical monachosorioid hair (one-, two-, or three-celled, club-shaped, or catenate unbranched hairs) on the frond and the sporangial stalk, and the lack of scales and true hairs on the rhizome (Table 3). Becari-Viana and Schwartsburd [41] carried out a detailed study of rhizome morphology and anatomy, and showed that the main rhizome type of Dennstaedtiaceae is long-creeping with alternate phyllotaxy and a solenostele, and while *Blotiella* and *Monachosorum* have ascending to short-creeping rhizomes with radial phyllotaxy and a dictyostele. Becari-Viana and Schwartsburd [41] proposed that the ancestor of Dennstaedtiaceae had a short-creeping to ascending rhizome. The solenostele evolved on the main lineage of Dennstaedtiaceae, while the dictyostele was primitive in *Monachosorum* (and was secondarily derived in *Blotiella*). All the morphological evidence indicated that *Monachosorum* occupies an isolated position, which also increases the difficulty in conclusively determining the position of *Monachosorum*.

**Table 3:** Comparison of major morphological characters of three subfamilies of Dennstaedtiaceae

	**Hypolepidoideae**	**Dennstaedtioideae**	**Monachosoroideae**
**Habitat**	edge-colonizer or thicket-forming of open habitats	edge-colonizer of open habitats	shade-tolerant
**Rhizomes**	long-creeping (ascending in *Blotiella*)	long-creeping	ascending to short-creeping
**Type of stele**	Solenostele (dictyostele in *Blotiella*)	solenostele	dictyostele
**Hairs**	multicellular hairs (bristles in *Paesia* and *Hypolepis*) on rhizome and fronds	multicellular hairs on rhizome and fronds	multicellular hairs on fronds, monachosorioid hairs (minute, few-celled, club-like, glandular hairs) on frond and sporangial stalk
Phyllotaxy of fronds	alternate (radial in *Blotiella*)	alternate	radial
Vascular bundles	one (*Paesia*) or several (*Pteridium*) vascular bundles; Omega-shaped or dissected C-shape (*Pteridium*);	Omega-shaped or C-shaped	two hypocampus bundles
Type of development of archegonia	common Leptosporangiate-type	common Leptosporangiate-type	almost simultaneous development of archegonia in the midst of several antheridia
**Sori**	marginal or submarginal	marginal or submarginal	terminal
**Indusia**	one (adaxial, outer) or two (abaxial and adaxial) indusia	one (adaxial) or two (abaxial and adaxial) indusia	absent
Spores	monolete (trilete in *Pteridium*)	trilete	trilete
Chromosome basic number (x)	24,26,28,29	30,31,33,34,40,42,43,46,47	56

Although the growing ecological conditions variously affect the prothalli of ferms, the salient morphological characteristics, such as the gross form and structure of the adult thallus, the nature of trichomes borne on the thallus, and the morphology of sex organs, the sequence of cell divisions at spore germination, the sequence of developmental stages, the type of development, are little altered [63]. The almost simultaneous development of archegonia in the midst of several antheridia in *Monachosorum* is a unique feature among the ferns [54], whereas that of Hypolepidoideae and Dennstaedtioideae is the common Leptosporangiate-type.

*Monachosorum maximowiczii* (Baker) Hayata is sometimes regarded as the independent genus *Ptilopteris* Hance based on its 1-pinnate and lanceolate fronds. The sister relationship between this species and members of *Monachosorum* was first confirmed by the chloroplast phylogeny [64]. In addition, nuclear *gap*Cp phylogeny by Ebihara et al. [65] showed that *M. maximowiczii* is a diploid parental species of *Monachosorum* × *flagellare* and *M. nipponicum*. In our analyses, *M. maximowiczii* and other members of *Monachosorum* began to diverge in the Miocene, and the latter was further differentiated in the Quaternary.

*Monachosorum maximowiczii* is the only known diploid species in the genus [65]. Hybridization occurs easily in mixed populations of the two parental species *M. maximowiczii* x *M. nipponicum* in Japan, and the hybrid taxon *Monachosorum* × *flagellare* (Maxim. ex Makino) Hayata is common and can produce irregularly shaped spores [65]. There are many common morphological characteristics between *Ptilopteris* and *Monachosorum*—terrestrial shade plants, erect or decumbent rhizomes, two hypocampus vascular bundles, thinly herbaceous leaves, monachosorioid hairs, sori with few (10 ~ 20) sporangia, exindusiate, and trilete spores. Considering the reticulate relationship [65], we prefer to place *M. maximowiczii* in *Monachosorum* rather than recognizing it as a separate genus *Ptilopteris*.

### The origin and diversification of suborder Dennstaedtiineae

The currently defined Dennstaedtiaceae (suborder Dennstaedtiineae) are estimated to have originated during the Jurassic and began to diversify during the Early Cretaceous, which is earlier than estimation from previous studies [34, 52, 66]. There is no doubt that a robust phylogenetic framework is foundationally important for molecular dating. However, intra-familial and intergeneric phylogenetic relationships in Dennstaedtiaceae have not been resolved or only weakly supported in these previous studies mentioned above. The lack of resolution has inhibited phylogenetic dating of Dennstaedtiaceae. Due to differences in phylogenetic relationships, the same fossil may be assigned as a calibration of different nodes. The fossils of *Krameropteris* (100.5 Ma) were used to calibrate the crown of *Monachosorum*-Hypolepidoideae by Schneider et al. [52], while they were calibrated at the crown of Dennstaedtiaceae in the study of Schwartsburd et al. [34].

Different interpretations of the strata and different placements of fossils in molecular dating can result in substantially different estimates of divergence times. The Late Cretaceous fossil genus *Microlepiopsis* Serbet & Rothwell was ascribed to Dennstaedtiaceae [67] based on solenostelic rhizomes, with sclerenchymatous pith and cortex and relatively simple frond trace anatomy. Eberth and Braman [68] revised the stratigraphy for the Horseshoe Canyon Formation of Canada, and described it as about 72 Ma. Both Schuettpelz and Pryer [66] and Testo and Sundue [27] used *Microlepiopsis* fossil with 70.6 Ma to calibrate the crown of Dennstaedtiaceae, and got different molecular ages—the former estimated Dennstaedtiaceae to have originated at about 166 Ma, while the latter estimated as about 240 Ma. Schwartsburd et al. [34] used the same fossil of *Microlepiopsis* with 80 Ma to calibrate the crown of Dennstaedtioideae-Hypolepidoideae (excluding *Monachosorum*), and Dennstaedtiaceae to have originated in the Early Cretaceous (135.78 Ma). The fossils of *Microlepiopsis* have not been incorporated in the present study with the consideration of their complicated stele types and anatomical heterogeneity of the vascular bundle (two U-shaped bundles that unite distally to form a W-shaped trace at the base of the stipe in *Microlepiopsis bramanii*; a C-shaped trace that diverges as a single bundle in *Microlepiopsis aulenbackii*) [67].

The BEAST analysis reveals a Jurassic origin of suborder Dennstaedtiineae in Asia, and the divergence of *Monachosorum* and other lineages of Dennstaedtiaceae occurred in the Early Cretaceous. The Late Jurassic-Early Cretaceous climatic conditions of higher rainfall and temperatures [69] favored the diversification of early-divergent shade-tolerant ancestors of suborder Dennstaedtiineae. With the increase of the continental arid belt and the decrease in global rainfall and temperature, the mid–high latitude fossil sites (e.g., Albert and Saskatchewan, Canada) became warmer during the mid-Cretaceous (95–70 Ma), which may have accelerated to the diversification of evolved with a change in habit: from shade-tolerant to edge-colonizing plants thriving in a warm climate in higher-latitude areas in this period.

*Microlepiopsis* fossils were found in the Horseshoe Canyon coal formation in Alberta, Canada, where the formation is unusually dry, producing no formation water in most parts, and the average water hydrostatic pressure is less than 30% [70]. After the mass extinction at the Cretaceous–Paleogene (K-Pg) boundary (66.0 Ma), Dennstaedtiaceae might have begun the second diversification in the Paleogene-Eocene to the Early Eocene. The second diversification of Dennstaedtiaceae occurred mainly during the Paleogene, and the divergence of Dennstaedtioideae and Hypolepidoideae was estimated to be at 60.88 and 45.5 Ma, respectively. The ancestors of Dennstaedtioideae-Hypolepidoideae may have become edge-colonizers with a long-creeping rhizome, and occupied new niches [34], which is very important to a disturbance-succession ecological regime associated with a Late Cretaceous wildfire regime, which possibly was responsible for its reorganization and higher diversification of Dennstaedtiaceae after the K-Pg event.

Some fern fossils, e.g., *Dennstaedtia americana* and *Dennastra sorimarginata* of Dennstaedtiaceae, *Onoclea hesperia* of Onocleaceae, *Woodwardia gravida* of Blechnaceae, were found in the early Paleocene (Danian) Ravenscrag Formation (Canada and USA; [71]). The Ravenscrag flora was considered to have been in the period when terrestrial ecosystems were recovering from a major shock of the K/Pg extinction [71]. Paleo-climatic and paleo-ecological studies of the Ravenscrag Butte flora indicated that the early Paleocene climate for southwestern Saskatchewan was warm, humid, with mild winters, and wet with some indication of precipitation seasonality, but without a pronounced dry season [71, 72]. The reconstructed climate of the Ravenscrag Butte flora is similar to modern coastal climates that exist near inland seas. The Ravenscrag Butte flora is most similar to contemporaneous fossil macrofloras from throughout western and northern North America, suggesting a potential consequence of vegetation reorganization after the K-Pg event [72]. There are also more recent fossils, from the Eocene (*Dennstaedtiopsis*, Canada and USA; [73]), Neogene, and even Quaternary [74, 75, 76]. All extant genera (and lineages) in Dennstaedtioideae and Hypolepidoideae were inferred to begin to diversify after the Late Oligocene.

### Elevating Monachosorum to the family rank

In the phylogenetic classification, monophyly is the primary principle and maximizing stability is the secondary principle for the recognition of taxa [3, 77]. A good example of this balance in the classification of ferns is the validation of Didymochlaenaceae. The phylogenetic analyses have resolved *Didymochlaena* Desv. as sister to the rest of the eupolypods I clade (the suborder Polypodiineae) [33, 45, 78] or sister to a clade comprising Dryopteridaceae and the DANLOPPT Clade [18]. Zhang and Zhang [33] evaluated the morphological characteristics that include sori, indusia, and spores of *Didymochlaena*, and found that *Didymochlaena* is distinct from the rest of eupolypods I in having elliptic-oblong sori, elongate indusia, and monolete spores with tuberculate and echinate on perispore. Zhang and Zhang [33] suggested the recognition of the family Didymochlaenaceae and validly described the family based on the principle of maximizing phylogenetic information—emphasizing distinct, deeply isolated lineages.

Differentiation time and diversity are other possible principles or criteria for taxon recognition in phylogenetic classifications. The divergence times of most families in polypods are estimated during the early Cretaceous (and even the late Jurassic) although a few families of eupolypods I clade seem to have differentiated slightly later and diverged from their closest sister group before the Paleogene [18]. *Monachosorum* (Monachosoroideae sensu Schwartsburd et al.) is a deeply isolated lineage from any other extant group of Dennstaedtiaceae. The divergence time estimates support the most recent common ancestor of Monachosoroideae and its closest relative dating back to the Early Cretaceous, which is consistent with the origin time of most families in polypod families.

*Monachosorum* is strongly supported as monophyletic with full support in our phylogenetic study. Based on the principle of monophyly for the recognition of taxa in phylogenetic classification, and the differentiation in genetic and morphological characteristics between Monachosoraceae and Dennstaedtiaceae, we argue for recognizing Monachosoraceae as a family in Polypodiales suborder Dennstaedtiineae. The remaining Dennstaedtiaceae become more homogeneous and easier to define (edge-colonizing or thicket-forming habit; long-creeping solenostelic rhizomes clothed with true hairs; marginal or submarginal sori; one or two indusia) (Table 3) when *Monachosorum* is segregated. The deep evolutionary divergence from its closest relatives and the shady habitat of *Monachosorum* also support that the genus belongs to a distinct family from Dennstaedtiaceae.

## Methods

### Taxon sampling, sequencing, and assembly

A total of 57 plastomes from suborder Dennstaedtiineae were sampled, consisting of 44 newly generated plastomes and the remaining 13 downloaded from GenBank (Table 4). Major lineages of Dennstaedtiaceae were represented by at least three species each (i.e., *Blotiella*, *Dennstaedtia*, *Histiopteris*, *Hiya*, *Hypolepis*, *Microlepia*, *Monachosorum*, *Paesia*, and *Pteridium*), and attention was paid to *Dennstaedtia* s.l. and *Monachosorum* to re-evaluate the phylogenetic status of these two genera based on plastomes. Additional 23 plastomes from Polypodiales suborder Pteridineae (10 taxa), suborder Aspleniineae (4 taxa), suborder Polypodiineae (4 taxa), suborder Lindsaeineae (4 taxa), and suborder Saccolomatineae (1 taxa) were employed as outgroups in the phylogenetic and dating analyses (Table 4).

**Table 4:** List of plastomes used in this study

Family name	Taxon	GenBank accession No
Dennstaedtiaceae	*Blotiella coursii* (Tardieu) Rakotondr. ex J.P. Roux	MT130655
Dennstaedtiaceae	*Blotiella lindeniana* (Hook.) R.M. Tryon	OP081137
Dennstaedtiaceae	*Blotiella madagascariensis* (Hook.) R.M. Tryon	OP081099
Dennstaedtiaceae	*Dennstaedtia ampla* (Baker) Bedd.	OP081098
Dennstaedtiaceae	*Dennstaedtia appendiculata* (Wall. ex Hook.) J. Sm.	OP081114
Dennstaedtiaceae	*Dennstaedtia glabrata* (Cesati) C.Chr.	OP081102
Dennstaedtiaceae	*Dennstaedtia globulifera* (Poir.) Hieron.	OP081133
Dennstaedtiaceae	*Dennstaedtia pilosella* (Hook.) Ching	MT130587
Dennstaedtiaceae	*Dennstaedtia punctilobula* (Michx.) T. Moore	OP081100
Dennstaedtiaceae	*Dennstaedtia samoensis* T. Moore	OP081119
Dennstaedtiaceae	*Dennstaedtia scabra* (Wall. ex Hook.) T. Moore	MT130669
Dennstaedtiaceae	*Dennstaedtia scandens* (Blume) T. Moore	OP081103
Dennstaedtiaceae	*Dennstaedtia tripinnatifida* Copel.	OP081120
Dennstaedtiaceae	*Dennstaedtia wilfordii* (T. Moore) Christ	OP081117
Dennstaedtiaceae	*Histiopteris herbacea* Copel.	OP081129
Dennstaedtiaceae	*Histiopteris incisa* (Thunberg) J. Smith	NC040220
Dennstaedtiaceae	*Histiopteris incisa* (Thunberg) J. Smith	MT130622
Dennstaedtiaceae	*Histiopteris stipulacea* Copel.	OP081108
Dennstaedtiaceae	*Hiya brooksiae* (Alderw.) H. Shang	OP081135
Dennstaedtiaceae	*Hiya brooksiae* (Alderw.) H. Shang	OP081126
Dennstaedtiaceae	*Hypolepis alpina* (Blume) Hook.	OP081110
Dennstaedtiaceae	*Hypolepis goetzei* Reimers	OP081125
Dennstaedtiaceae	*Hypolepis punctata* (Thunb.) Mett. ex Kuhn	OP081123
Dennstaedtiaceae	*Hypolepis punctata* (Thunb.) Mett. ex Kuhn	MT130616
Dennstaedtiaceae	*Hypolepis repens* (L.) C. Presl	OP081118
Dennstaedtiaceae	*Leptolepia novae-zelandiae* (Colenso) Mett. ex Diels	OP081136
Dennstaedtiaceae	*Microlepia herbacea* Ching & C.Chr. ex C.Chr. & Tardieu	OP081107
Dennstaedtiaceae	*Microlepia khasiyana* (Hook.) C. Presl	MT130633
Dennstaedtiaceae	*Microlepia marginata* (Panzer) C. Christensen	MT130649
Dennstaedtiaceae	*Microlepia obtusiloba* Hayata	MT130570
Dennstaedtiaceae	*Microlepia platyphylla* (D. Don) J. Smith	MT130685
Dennstaedtiaceae	*Microlepia speluncae* (L.) T. Moore	OP081121
Dennstaedtiaceae	*Microlepia speluncae* (L.) T. Moore	OP081101
Dennstaedtiaceae	*Microlepia speluncae* (L.) T. Moore	OP081106
Dennstaedtiaceae	*Microlepia tenera* Christ	OP081122
Dennstaedtiaceae	*Microlepia trapeziformis* (Roxb.) Kuhn	OP081128
Dennstaedtiaceae	*Microlepia yaoshanica* Ching	OP081097
Dennstaedtiaceae	*Monachosorum arakii* Tagawa	OP081116
Dennstaedtiaceae	*Monachosorum flagellare* (Maxim. ex Makino) Hayata	OP081127
Dennstaedtiaceae	*Monachosorum henryi* Christ	MT130593
Dennstaedtiaceae	*Monachosorum henryi* Christ	OP081132
Dennstaedtiaceae	*Monachosorum henryi* Christ	OP081138
Dennstaedtiaceae	*Monachosorum henryi* Christ	OP081105
Dennstaedtiaceae	*Monachosorum henryi* Christ	OP081112
Dennstaedtiaceae	*Monachosorum maximowiczii* (Baker) Hayata	OP081109
Dennstaedtiaceae	*Monachosorum nipponicum* Makino	OP081111
Dennstaedtiaceae	*Monachosorum nipponicum* Makino	OP081131
Dennstaedtiaceae	*Monachosorum subdigitatum* (Blume) Kuhn	OP081130
Dennstaedtiaceae	*Monachosorum subdigitatum* (Blume) Kuhn	OP081124
Dennstaedtiaceae	*Oenotrichia maxima* (E. Fourn.) Copel.	OP081113
Dennstaedtiaceae	*Paesia elmeri* Copel.	OP081115
Dennstaedtiaceae	*Paesia elmeri* Copel.	OP081104
Dennstaedtiaceae	*Paesia elmeri* Copel.	OP081134
Dennstaedtiaceae	*Paesia glandulosa* (Sw.) Kuhn	OP081139
Dennstaedtiaceae	*Pteridium aquilinum* (L.) Kuhn	NC014348
Dennstaedtiaceae	*Pteridium caudatum* (L.) Maxon	OP081096
Dennstaedtiaceae	*Pteridium revolutum* (Blume) Nakai	MT130625
OUTGROUPS		
Cystodiaceae	*Cystodium sorbifolium* (Sm.) J. Sm.	MT130630
Cystopteridaceae	*Acystopteris japonica* (Luerss.) Nakai	MT130696
Diplaziopsidaceae	*Diplaziopsis brunoniana* (Wall.) W.M. Chu	MT130567
Dryopteridaceae	*Dryopteris crassirhizoma* Nakai	MT130689
Hypodematiaceae	*Hypodematium crenatum* (Forssk.) Kuhn & Decken	MT130540
Lindsaeaceae	*Lindsaea cultrata* (Willd.) Sw.	MT130672
Lindsaeaceae	*Osmolindsaea odorata* (Roxb.) Lehtonen & Christenh	MT130576
Lonchitidaceae	*Lonchitis hirsute* L.	MT130654
Nephrolepidaceae	*Nephrolepis cordifolia* (L.) C. Presl	MT130673
Oleandraceae	*Oleandra wallichii* (Hook.) C. Presl	MT130650
Pteridaceae	*Acrostichum aureum* L.	MT130571
Pteridaceae	*Adiantum hispidulum* Sw.	LT615217
Pteridaceae	*Adiantum shastense* Huiet & A.R. Sm.	NC037478
Pteridaceae	*Calciphilopteris ludens* (Wall. ex Hook.) Yesilyurt & H. Schneid.	MT130590
Pteridaceae	*Ceratopteris pteridoides* (Hook.) Hieron.	MT130583
Pteridaceae	*Cheilanthes micropteris* Sw.	MH173078
Pteridaceae	*Cryptogramma acrostichoides* R. Br.	MH173081
Pteridaceae	*Llavea cordifolia* Lag.	MH173088
Pteridaceae	*Onychium japonicum* var*. lucidum* (D. Don) Christ	MT130597
Pteridaceae	*Pteris vittata* L.	MT130668
Saccolomataceae	*Saccoloma elegans* Kaulf.	MT130580
Thelypteridaceae	*Macrothelypteris torresiana* (Gaud.) Ching	MT130591
Woodsiaceae	*Woodsia manchuriensis* Hook.	MT130698

Leaf material was collected from living plants in the field, and herbarium specimens (CSH, KUN, MO, TAIF, TNS, and WELT). DNA extraction, library preparation, and Illumina sequencing were conducted following the protocol of plastome sequencing from herbarium specimens [79]. The libraries were then sequenced on Illumina Hiseq 2500 or X-Ten sequencing system (Illumina Inc.) to generate 150 bp paired-end reads, with *ca*. 1–3 Gb raw data for each sample. DNA extraction and library preparation were conducted at the Molecular Biology Experiment Center, Germplasm Bank of Wild Species, Kunming Institute of Botany (CAS), and Illumina sequencing was conducted at BGI Genomics Co., Ltd (Shenzhen, China). De novo assemblies were constructed with GetOrganelle toolkits [80]. Reference-guided connection and gene annotation were conducted using Bandage 0.8.1 [81] and Geneious 9.1.4 [82], using previously published fern plastomes as references. The voucher information, the plastome characteristics, and the GenBank accession number of newly sequenced samples are provided in Table 4 and Additional file 1.

### Data matrices and phylogenetic analyses

We utilized different data sets, including the complete plastid genome (plastome) sequences (pt) and the coding region of the 86 protein-coding genes (CDS) to conduct the phylogenetic reconstruction. All sampled plastomes were aligned using MAFFT [83], and the unreliably aligned regions were filtered using Gblocks v0.91b [84] with default parameters except that all gap positions were allowed. The Pt data set (the main data set) has an aligned length of 124,738 bp and an average GC content of 42.7%. We also constructed a sub-dataset consisting of all 86 coding sequences (CDS data set), which has an aligned length of 72,828 bp and an average GC content of 43% (Table 2).

We performed Maximum Likelihood (ML) analyses and Bayesian Inferences (BI) on the main data set (pt) and the CDS data set. The heterogeneous GHOST [85] model GTR + H4 were used in ML analyses of the main data set, and the gene-partitioned model estimated by PartitionFinder2 [86] were used in ML analyses of the CDS data set. ML analyses were conducted using IQ-tree 1.6.12 [87] with support values estimated by 10,000 ultrafast bootstrap replicates. Bayesian inferences were conducted using MrBayes 3.2.6 [88], and with the GTR + I + G substitution model. Five-million-generation iterations with trees being sampled per 1,000 generations, two runs with four chains were performed in parallel. The MCMC (Markov chain Monte Carlo) output was examined to check convergence and to ensure that all the ESS (effective sample sizes) values were above 500.

### Divergence time estimation

We estimated the divergence times of Polypodiales suborder Dennstaedtiineae based on the main pt data set. Bayesian estimations of divergence times were conducted with BEAST v.2.6.6 [89], using an un-partitioned GTR + I + G nucleotide substitution model, birth–death tree prior and lognormal uncorrelated relaxed clock model, and the phylogeny from our ML analysis as the starting tree, and four fossil calibrations (two from Dennstaedtiaceae and two from Pteridaceae) were adopted in the dating analyses.

The oldest fossils of Dennstaedtiaceae were found in the Lower Cretaceous of Kachin, Myanmar (100.5 Ma, Late Albian, early Late Cretaceous [52]). The fossils of *Krameropteris resinatus* H. Schneid., A. R. Schmidt & Heinrichs were unequivocally assigned to the early diverging Dennstaedtiaceae based on its polypod sporangia with trilete spores, exindusiate sori, and free branched veins. The fossils of *Krameropteris* (100.5 Ma) were used to calibrate the crown of Dennstaedtiaceae (the split between *Monachosorum* and the aggregate of Dennstaedtioideae-Hypolepidoideae). The early Eocene fossils of *Dennstaedtia christophelii* (48.9 Ma) [75] were used to calibrate the crown of Dennstaedtioideae (*Dennstaedtia s.l.*) based on its cuplike indusial, marginal and round sori, a vertical and interrupted annulus, and once-pinnate-pinnatifid to bipinnate lamina. The combination of characteristics of fossil *Heinrichsia cheilanthoides* L. Regalado, A.R. Schmidt, M. Krings & H. Schneid. (100.5 Ma, Kachin, Myanmar; [90]), tetrahedral-globose trilete spores and marginal sori protected by reflexed pseudo-indusia, is strong support of the fossil's affinities with Pteridaceae. This fossil was adopted to calibrate the crown of Pteridaceae. The Maastrichtian *Acrostichum* fossil (66 Ma, [91]) was used to constrain the divergence between Parkerioideae (including *Acrostichum* and *Ceratopteris*) and Pteridoideae. All fossil ages were used as an offset in the lognormal priors (mean: 3.0, SD: 1.0). Furthermore, the estimated divergence times of the Polypodiales crown (95% HPD: 191.62–219.87 Ma, [18]) were employed as the minimum–maximum age constraints (uniform prior) of secondary molecular root calibrations. One run of 10 billion generations was conducted, sampling every 2,000 generations. Convergence was attained within two to five billion generations, and the ESS values for all parameters were over 200. We removed the first five billion generations as burn-in, and used the remaining *ca*. 250 thousand trees to generate the maximum clade credibility tree (MCC) by TreeAnnotator with a posterior probability limit of 0.5 and median node heights.

## Supplementary Information

**Additional file 1.** Information of Voucher specimens, the characteristics, and GenBank accession numbers of newly generated plastomes in this study.

**Additional file 2.** Maximum likelihood (ML) phylogeny of Polypodiales suborder Dennstaedtiineae based on CDS data set.

**Additional file 3.** Bayesian inference (BI) phylogeny of Polypodiales suborder Dennstaedtiineae based on CDS data set.

## Data Availability

The complete chloroplast genomes generated in this study were submitted to the NCBI database (https://www.ncbi.nlm.nih.gov/) with GenBank accession numbers from OP081096 to OP081139. All other data and material analyzed in the current study are included in the manuscript and the supplementary information files.

## References

[1] Takhtajan AL (1953). Phylogenetic principles of the system of higher plants. Bot Rev.

[2] Smith AR, Pryer KM, Schuettpelz E, Korall P, Schneider H, Wolf PG (2006). A classification for extant ferns. Taxon.

[3] PPG (The Pteridophyte Phylogeny Group) I (2016). A community-derived classification for extant lycophytes and ferns. J Syst Evol.

[4] Chen DK, Zhou XM, Rothfels CJ, Shepherd LD, Knapp R, Zhang L, Lu NT, Fan XP, Wan X, Gao XF, He H, Zhang LB (2022). A global phylogeny of Lycopodiaceae (Lycopodiales; lycophytes) with the description of a new genus, *Brownseya*, from Oceania. Taxon.

[5] Wei R, Zhang XC (2022). A revised subfamilial classification of Polypodiaceae based on plastome, nuclear ribosomal, and morphological evidence. Taxon.

[6] Wei R, Yang J, He LJ, Liu HM, Hu JY, Liang SQ, Wei XP, Zhao CF, Zhang XC (2021). Plastid phylogenomics provides novel insights into the infrafamilial relationship of Polypodiaceae. Cladistics.

[7] Zhang L, Zhou XM, Lu NT, Zhang LB (2017). Phylogeny of the fern subfamily Pteridoideae (Pteridaceae; Pteridophyta), with the description of a new genus: *Gastoniella*. Mol Phylogenet Evol.

[8] Fawcett S, Smith AR, Sundue M, Burleigh JG, Sessa EB,  Kuo LY, Chen CW, Testo WL, Kessler M, Consortium G, Barrington DS (2021). A global phylogenomic study of the Thelypteridaceae. Syst Bot.

[9] Kuo LY, Chang YH, Huang YH, Testo W, Ebihara A, Rouhan G, Quintanilla LG, Watkins JE, Huang YM, Li FW (2020). A global phylogeny of *Stegnogramma* ferns (Thelypteridaceae): generic and sectional revision, historical biogeography and evolution of leaf architecture. Cladistics.

[10] Zhou XM, Zhang L, Lu NT, Gao XF, Zhang LB (2018). Pteridryaceae: a new fern family of Polypodiineae (Polypodiales) including taxonomic treatments. J Syst Evol.

[11] Ponce MM, Scataglini MA (2022). Phylogenetic position of South American *Cheilanthes* (Cheilanthoideae, Pteridaceae): advances in the generic circumscription and segregation of the new genus *Mineirella*. J Syst Evol.

[12] Zhang L, Zhou XM, Liang ZL, Fan XP, Lu NT, Song MS, Knapp R, Gao XF, Sun H, Zhang LB (2020). Phylogeny and classification of the tribe Lepisoreae (Polypodiaceae; pteridophyta) with the description of a new genus, *Ellipinema* gen. nov., segregated from* Lepisorus*. Mol Phylogenet Evol.

[13] Zhang L, Fan XP, Petchsri S, Zhou L, Pollawatn R, Zhang X, Zhou XM, Lu NT, Knapp R, Chantanaorrapint S, Limpanasittichai P, Sun H, Gao XF, Zhang LB (2020). Evolutionary relationships of the ancient fern lineage the adder's tongues (Ophioglossaceae) with description of *Sahashia* gen. nov. Cladistics.

[14] Liu HM, Rakotondrainibe F, Hennequin S, Schneider H (2020). The significance of *Rouxopteris* (Gleicheniaceae, Polypodiopsida): a new genus endemic to the Madagascan region. Plant Syst Evol.

[15] George LO, Pryer KM, Kao TT, Huiet L, Windham MD (2019). *Baja*: a new monospecific genus segregated from *Cheilanthes* s. l. (Pteridaceae). Syst Bot.

[16] Almeida TE, Salino A, Dubuisson JY, Hennequin S (2017). *Adetogramma* (Polypodiaceae), a new monotypic fern genus segregated from *Polypodium*. Phytokeys.

[17] Du XY, Kuo LY, Zuo ZY, Li DZ, Lu JM (2022). Structural variation of plastomes provides key insight into the deep phylogeny of ferns. Front Plant Sci.

[18] Du XY, Lu JM, Zhang LB, Wen J, Kuo LY, Mynssen MC, Schneider H, Li DZ (2021). Simultaneous diversification of Polypodiales and angiosperms in the Mesozoic. Cladistics.

[19] Chen CW, Sundue M, Kuo LY, Teng WC, Huang YM (2017). Phylogenetic analyses place the monotypic *Dryopolystichum* within Lomariopsidaceae. Phytokeys.

[20] Chen CW, Lindsay S, Kuo LY, Fraser-Jenkins CR, Ebihara A, Luu HT, Park CW, Chao YS, Huang YM, Chiou WL (2017). A systematic study of East Asian vittarioid ferns (Pteridaceae: Vittarioideae). Bot J Linn Soc.

[21] Engels ME, Canestraro BK (2017). x *Cyclobotrya*: a new hybrid genus between *Cyclodium* and *Polybotrya* (Dryopteridaceae) from the Brazilian Amazon. Brittonia.

[22] Lu NT, Zhou XM, Zhang L, Knapp R, Li CX, Fan XP, Zhou L, Wei HJ, Lu JM, Xu B, Peng YL, Gao XF, Zhang LB (2020). A global plastid phylogeny of the cliff fern family Woodsiaceae and a two-genus classification of Woodsiaceae with the description of x *Woodsimatium* nothogen. nov. Taxon.

[23] Liu HM, Schuettpelz E, Schneider H (2020). Evaluating the status of fern and lycophyte nothotaxa in the context of the Pteridophyte Phylogeny Group classification (PPG I). J Syst Evol.

[24] Pryer KM, Schuettpelz E, Wolf PG, Schneider H, Smith AR, Cranfill R (2004). Phylogeny and evolution of ferns (monilophytes) with a focus on the early leptosporangiate divergences. Amer J Bot.

[25] Qi XP, Kuo LY, Guo CC, Li H, Li ZY, Qi J, Wang LB, Hu Y, Xiang JY, Zhang CF, Guo J, Huang CH, Ma H (2018). A well-resolved fern nuclear phylogeny reveals the evolution history of numerous transcription factor families. Mol Phylogen Evol.

[26] Schuettpelz E, Pryer KM (2007). Fern phylogeny inferred from 400 leptosporangiate species and three plastid genes. Taxon.

[27] Testo W, Sundue M (2016). A 4000-species dataset provides new insight into the evolution of ferns. Mol Phylogen Evol.

[28] Ching RC (1978). The Chinese fern families and genera: systematic arrangement and historical origin. Acta Phytotaxon Sin.

[29] Christenhusz MJM, Zhang XC, Schneider H (2011). A linear sequence of extant families and genera of lycophytes and ferns. Phytotaxa.

[30] Christenhusz MJM, Schneider H (2011). Corrections to Phytotaxa 19: linear sequence of lycophytes and ferns. Phytotaxa.

[31] Liu HM, Jiang RH, Guo J, Hovenkamp P, Perrie LR, Shepherd L, Hennequin S, Schneider H (2013). Towards a phylogenetic classification of the climbing fern genus *Arthropteris*. Taxon.

[32] Mynssen CM, Vasco A, Moran RC, Sylvestre LS, Rouhan G (2016). Desmophlebiaceae and *Desmophlebium*: a new family and genus of eupolypod II ferns. Taxon.

[33] Zhang LB, Zhang L (2015). Didymochlaenaceae: a new fern family of eupolypods I (Polypodiales). Taxon.

[34] Schwartsburd PB, Perrie LR, Brownsey P, Shepherd LD, Sundue MA (2020). New insights into the evolution of the fern family Dennstaedtiaceae from an expanded molecular phylogeny and morphological analysis. Mol Phylogenet Evol.

[35] Shang H, Sundue M, Wei R, Wei XP, Luo JJ, Liu L, Schwartsburd PB, Yan YH, Zhang XC (2018). *Hiya*: a new genus segregate from Hypolepis in the fern family Dennstaedtiaceae, based on phylogenetic evidence and character evolution. Mol Phylogen Evol.

[36] Yan YH, Qi XP, Liao WB, Xing FW, Ding MY, Wang FG, Zhang XC, Wu ZH, Serizawa S, Prado J, Funston AM, Gilbert MG, Nooteboom HP. Dennstaedtiaceae, 147–168. In: Wu ZY, Raven PH, Hong, DY, (Eds.). Flora of China. vol. 2–3 (Pteridophytes). Science Press, Beijing; Missouri Botanical Garden Press, St. Louis; 2013.

[37] Pichi Sermolli REG (1970). Fragmenta Pteridologiae—II. Webbia.

[38] Kramer KU. Dennstaedtiaceae. In: Kramer KU, Green PS (Eds.), Vol. I. Pteridophytes and Gymnosperms, 81–94. In: Kubitzki K (ed.) The families and genera of vascular plants. Springer-Verlag Heidelberg, Germany; 1990.

[39] Wolf PG (1995). Phylogenetic analyses of *rbc*L and nuclear ribosomal RNA gene sequences in Dennstaedtiaceae. Amer Fern J.

[40] Perrie LR, Shepherd LD, Brownsey PJ (2015). An expanded phylogeny of the Dennstaedtiaceae ferns: *Oenotrichia* falls within a non-monophyletic *Dennstaedtia*, and *Saccoloma* is polyphyletic. Aust Syst Bot.

[41] Becari-Viana I, Schwartsburd PB (2017). Morpho-anatomical studies and evolutionary interpretations of the rhizomes of extant Dennstaedtiaceae. Amer Fern J.

[42] Kramer KU. Monachosoraceae. In: Kramer KU, Green PS (Eds.), Vol. I. Pteridophytes and Gymnosperms, 187–188. In: Kubitzki K (ed.) The families and genera of vascular plants. Springer-Verlag Heidelberg, Germany; 1990.

[43] Ching RC (1940). On natural classification of the family “Polypodiaceae”. Sunyatsenia.

[44] Nayar BK (1970). A phylogenetic classification of the homosporous ferns. Taxon.

[45] Kuo LY, Li FW, Chiou WL, Wang CN (2011). First insights into fern *mat*K phylogeny. Mol Phylogen Evol.

[46] Lu JM, Zhang N, Du XY, Wen J, Li DZ (2015). Chloroplast phylogenomics resolves key relationships in ferns. J Syst Evol.

[47] Rothfels CJ, Li FW, Sigel EM, Huiet L, Larsson A, Burge DO, Ruhsam M, Deyholos M, Soltis DE, Stewart CN, Shaw SW, Pokorny L, Chen T, dePamphilis C, DeGironimo L, Chen L, Wei XF, Sun X, Korall P, Stevenson DW, Graham SW, Wong GKS, Pryer KM (2015). The evolutionary history of ferns inferred from 25 low-copy nuclear genes. Am J Bot.

[48] Liu HM (2016). Embracing the pteridophyte classification of Ren-Chang Ching using a generic phylogeny of Chinese ferns and lycophytes. J Syst Evol.

[49] Schölch A (2000). Relations between submarginal and marginal sori in ferns I. The sori of selected Hypolepidaceae and Dennstaedtiaceae. Plant Syst. Evol.

[50] Schölch A (2003). Relations between submarginal and marginal sori in ferns III. Superficial sori with emphasis on Pteridaceae and morphological relations to marginal sori. Plant Syst Evol.

[51] Hernández-Hernández V, Terrazas T, Mehltreter K, Angeles G (2012). Studies of petiolar anatomy in ferns: structural diversity and systematic significance of the circumendodermal band. Bot J Linn Soc.

[52] Schneider H, Schmidt AR, Heinrichs J (2016). Burmese amber fossils bridge the gap in the cretaceous record of polypod ferns. Perspect Plant Ecol.

[53] Triana-Moreno LA, Schwartsburd PB, Yañez A, Pena NTL, Kuo LY, Rothfels C, Sundue M (2022). (2892) Proposal to conserve the name *Dennstaedtia* (Dennstaedtiaceae) with a conserved type. Taxon.

[54] Nair GB, Sen U (1974). Morphology and anatomy of *Monachosorum subdigitatum* (Bl.) Kuhn with a discussion on its affinities. Ann Bot-London.

[55] Tagawa M (1937). *Monachosorum* and *Ptilopteris*. Jpn J Bot.

[56] Lovis JD (1977). Evolutionary patterns and processes in ferns. Adv Bot Res.

[57] Pichi Sermolli REG (1977). Tentamen Pteridophytorum genera in taxonomicum ordinem redigendi. Webbia.

[58] Tryon RM, Tryon AF (1982). Ferns and fern allies, with special reference to tropical America.

[59] Christensen C, Verdoorn F (1938). Filicinae. Manual of pteridology.

[60] Holttum RE (1947). A revised classification of leptosporangiate ferns. Bot J Linn Soc.

[61] Copeland EB (1947). Genera Filicum.

[62] Mickel JT. The classification and phylogenetic position of the Dennstaedtiaceae. In: Jermy AC, Crabbe JA, Thomas BA (Eds.), The Phylogeny and Classification of the Ferns. Bot J Linn Soc 1973;67(supp. 1):135–144.

[63] Nayar BK, Kaur S (1971). Gametophytes of homosporous ferns. Bot Rev.

[64] Ebihara A (2011). *rbc*L phylogeny of Japanese pteridophyte flora and implications on infrafamilial systematics. Bull Natl Mus Nat Sci Ser B.

[65] Ebihara A, Nakato N, Amoroso VB, Hidayat A, Kuo LY (2016). *Monachosorum arakii* Tagawa (Dennstaedtiaceae) is a relict "international" hybrid: a reassessment of the *Monachosorum* species. Syst Bot.

[66] Schuettpelz E, Pryer KM (2009). Evidence for a Cenozoic radiation of ferns in an angiosperm-dominated canopy. Proc Natl Acad Sci USA.

[67] Serbet R, Rothwell GW (2003). Anatomically preserved ferns from the late cretaceous of western North America: Dennstaedtiaceae. Intl J Pl Sci.

[68] Eberth DA, Braman DR (2012). A revised stratigraphy and depositional history for the horseshoe canyon formation (Upper Cretaceous), southern Alberta plains. Can J Earth Sci.

[69] Chaboureau AC, Sepulchre P, Donnadieu Y, Franc A (2014). Tectonic-driven climate change and the diversification of angiosperms. Proc Natl Acad Sci USA.

[70] Hoch OF (2005). The dry coal anomaly-the Horseshoe canyon formation of Alberta, Canada.

[71] McIver EE, Basinger JF (1993). Flora of the Ravenscrag Formation (Paleocene), Southwestern Saskatchewan. Canada Palaeontographica Canadiana.

[72] West CK, Reichgelt T, Basinger JF (2021). The Ravenscrag Butte flora: Paleoclimate and paleoecology of an early Paleocene (Danian) warm-temperate deciduous forest near the vanishing inland Cannonball Seaway. Palaeogeogr Palaeocl.

[73] Shi CS, Schopf JW, Kudryavtsev AB (2013). Characterization of the stem anatomy of the Eocene fern *Dennstaedtiopsis aerenchymata* (Dennstaedtiaceae) by use of confocal laser scanning microscopy. Amer J Bot.

[74] Collinson ME (2001). Cainozoic ferns and their distribution. Brittonia.

[75] Pigg KB, DeVore ML, Greenwood DR, Sundue M, Schwartsburd P, Basinger JF (2021). Fossil Dennstaedtiaceae and Hymenophyllaceae from the Early Eocene of the Pacific Northwest. Int J Pl Sci.

[76] Fossilworks. Available from: http://fossilworks.org/. 2022. Accessed on Apr 2022.

[77] Backlund A, Bremer K (1998). To be or not to be–principles of classification and monotypic plant families. Taxon.

[78] Lehtonen S, Wahlberg N, Christenhusz MJM (2012). Diversification of lindsaeoid ferns and phylogenetic uncertainty of early polypod relationships. Bot J Linn Soc.

[79] Zeng CX, Hollingsworth PM, Yang J, He ZS, Zhang ZR, Li DZ, Yang JB (2018). Genome skimming herbarium specimens for DNA barcoding and phylogenomics. Plant Methods.

[80] Jin JJ, Yu WB, Yang JB, Song Y, dePamphilis CW, Yi TS, Li DZ (2020). GetOrganelle: a fast and versatile toolkit for accurate de novo assembly of organelle genomes. Genome Biol.

[81] Wick RR, Schultz MB, Zobel J, Holt KE (2015). Bandage: interactive visualization of de novo genome assemblies. Bioinformatics.

[82] Kearse M, Moir R, Wilson A, Stones-Havas S, Cheung M, Sturrock S, Buxton S, Cooper A, Markowitz S, Duran C, Thierer T, Ashton B, Meintjes P, Drummond A (2012). Geneious Basic: an integrated and extendable desktop software platform for the organization and analysis of sequence data. Bioinformatics.

[83] Katoh K, Kuma K, Toh H, Miyata T (2005). MAFFT version 5: improvement in accuracy of multiple sequence alignment. Nucleic Acids Res.

[84] Talavera G, Castresana J (2007). Improvement of phylogenies after removing divergent and ambiguously aligned blocks from protein sequence alignments. Syst Biol.

[85] Crotty SM, Minh BQ, Bean NG, Holland BR, Tuke J, Jermiin LS, Haeseler AV (2020). GHOST: recovering historical signal fromheterotachously evolved sequence alignments. Syst Biol.

[86] Lanfear R, Frandsen PB, Wright AM, Senfeld T, Calcott B (2017). PartitionFinder 2: new methods for selecting partitioned models of evolution for molecular and morphological phylogenetic analyses. Mol Biol Evol.

[87] Nguyen LT, Schmidt HA, von Haeseler A, Minh BQ (2015). IQ-TREE: a fast and effective stochastic algorithm for estimating maximum-likelihood phylogenies. Mol Biol Evol.

[88] Ronquist F, Teslenko M, van der Mark P, Ayres DL, Darling A, Höhna S, Larget B, Liu L, Suchard MA, Huelsenbeck JP (2012). MrBayes 3.2: Efficient Bayesian phylogenetic inference and model choice across a large model space. Syst Biol.

[89] Drummond AJ, Suchard MA, Xie D, Rambaut A (2012). Bayesian phylogenetics with BEAUti and the BEAST 1.7. Mol Biol Evol.

[90] Regalado L, Schmidt AR, Müller P, Niedermeier L, Krings M, Schneider H. *Heinrichsia cheilanthoides* gen. et sp. nov., a fossil fern in the family Pteridaceae (Polypodiales) from the Cretaceous amber forests of Myanmar. J Syst Evol. 2019;57:329–38.

[91] Bonde SD, Kumaran KPN (2002). The oldest macrofossil record of the mangrove fern *Acrostichum* L. from the late cretaceous deccan intertrappean beds of India. Cretaceous Res.

