# Supplementary material for: Plastome phylogenomic analysis reveals evolutionary divergences of Polypodiales suborder Dennstaedtiineae
Source: BMC Plant Biol. 2022 Nov 2;22:511. doi: 10.1186/s12870-022-03886-1 (PMC9628275; doi:10.1186/s12870-022-03886-1)
Supplement: Supplementary file 1 — Additional file 1. Information of Voucher specimens, the characteristics, and GenBank accession numbers of newly generated plastomes in this study. [file 12870_2022_3886_MOESM1_ESM.pdf]

Additional file 1. Information of Voucher specimens, the characteristics, and GenBank accession numbers of newly generated plastomes in this study.

Taxon	Voucher (Herbarium)	Locality	Size (bp)	GC %	GenBank No.
Blotiella lindeniana (Hook.) R.M. Tryon	Alexander Rojas 8327 (MO)	Guanacaste, Costo Rica	150,940	42.9%	OP081137
Blotiella madagascariensis (Hook.) R.M. Tryon	Rakotovao C 5895(MO)	Moramanga, Madagascar	151,103	43.2%	OP081099
Dennstaedtia ampla (Baker) Bedd.	Wade 4671(TAIF)	Sabah, Malaysia	149,728	42.1%	OP081098
Dennstaedtia appendiculata (Wall. ex Hook.) J. Sm.	Zuo2600(KUN)	Xizang, China	148,429	44.1%	OP081114
Dennstaedtia glabrata (Cesati) C.Chr.	Kuo 1926(TAIF)	Quezon, the Philippines	149,669	42.1%	OP081102
Dennstaedtia globulifera (Poir.) Hieron.	Maarten MJ Christenhusz 4745(MO)	Santa Catarina, Brazil	150,846	41.8%	OP081133
Dennstaedtia punctilobula (Michx.) T. Moore	Daniel Atha 6350(MO)	New York, USA	148,118	44.1%	OP081100
Dennstaedtia samoensis T. Moore	Wade 3032(TAIF)	Rendova, Solomon Islands	150,638	42.3%	OP081119
Dennstaedtia scandens (Blume) T. Moore	Takeuchi WN 7422(MO)	New Guinea	149,795	42.1%	OP081103
Dennstaedtia tripinnatifida Copel.	Wade 4441(TAIF)	Makira, Solomon Islands	149,696	42.0%	OP081120
Dennstaedtia wilfordii (T. Moore) Christ	Zuo1950(KUN)	Jiuzhaigou, Sichuan, China	148,426	44.1%	OP081117
Histiopteris herbacea Copel.	Wade 2830(TAIF)	Ranongga, Solomon Islands	158,547	42.9%	OP081129
Histiopteris stipulacea Copel.	Kato M et al 14177(MO)	Kecamatan, Indonesia	153,225	42.3%	OP081108
Hiya brooksiae (Alderw.) H. Shang	SG1731(CSH)	CSH	154,584	43.2%	OP081135
Hiya brooksiae (Alderw.) H. Shang	Wade 4710(TAIF)	Sabah, Malaysia	152,766	43.1%	OP081126
Hypolepis alpina (Blume) Hook.	Kuo 2706(TAIF)	Mindano, the Philippines	150,844	42.3%	OP081110
Hypolepis goetzei Reimers	Peris Kamau 483(MO)	Mount Kenya, Kenya	150,821	42.4%	OP081125
Hypolepis punctata (Thunb.) Mett. ex Kuhn	Gardner RO 5110(MO)	Stratford, New Zealand	151,636	42.5%	OP081123
Hypolepis repens (L.) C. Presl	Jimenez I 646(MO)	Huehuetenango, Guatemala	152,544	42.4%	OP081118
Leptolepia novae-zelandiae (Colenso) Mett. ex Diels	Gardner RO 2443(MO)	Kaitarakihi, New Zealand	147,263	42.4%	OP081136
Microlepia herbacea Ching & C.Chr. ex C.Chr. & Tardieu	Wade 2653(TAIF)	Lam Dong, Vietnam	151,018	43.7%	OP081107
Microlepia speluncae (L.) T. Moore	Jongkind CCH 10557(MO)	Nzerekore, Guinea	151,710	43.5%	OP081121

**Taxon**	**Voucher (Herbarium)**	**Locality**	**Size (bp)**	**GC %**	**GenBank No.**
Microlepia speluncae (L.) T. Moore	Kuo 1950(TAIF)	Quezon, the Philippines	151,569	43.6%	OP081101
Microlepia speluncae (L.) T. Moore	Kuo 2775(TAIF)	Mindano, the Philippines	151,055	43.5%	OP081106
Microlepia tenera Christ	Zuo3206(KUN)	Yunnan, China	149,024	43.3%	OP081122
Microlepia trapeziformis (Roxb.) Kuhn	Tsi zhanhuo 91-24(MO)	Yunnan, China	151,149	43.7%	OP081128
Microlepia yaoshanica Ching	Zuo1256(KUN)	Yunnan, China	149,914	43.6%	OP081097
Monachosorum arakii Tagawa	Nakato2867(TNS)	Hyogo, Japan	152,138	45.1%	OP081116
Monachosorum flagellare (Maxim. ex Makino) Hayata	Zuo4073(KUN)	Fujian, China	152,137	45.1%	OP081127
Monachosorum henryi Christ	DLJ2019263(KUN)	Yunnan, China	151,998	45.0%	OP081132
Monachosorum henryi Christ	Zuo1276(KUN)	Yunnan, China	151,951	45.0%	OP081138
Monachosorum henryi Christ	Zuo1561(KUN)	Yunnan, China	152,064	45.1%	OP081105
Monachosorum henryi Christ	Zuo2657(KUN)	Xizang, China	151,947	45.0%	OP081112
Monachosorum maximowiczii (Baker) Hayata	Tsugaru S & Takahashi T 26003(MO)	Kyoto, Japan	150,616	45.5%	OP081109
Monachosorum nipponicum Makino	AE3284(TNS)	Wakayama, Japan	152,129	45.1%	OP081111
Monachosorum nipponicum Makino	EI060523-2(TNS)	Saitama, Japan	152,183	45.1%	OP081131
Monachosorum subdigitatum (Blume) Kuhn	Kuo 2756(TAIF)	Mindano, the Philippines	151,212	45.0%	OP081130
Monachosorum subdigitatum (Blume) Kuhn	Kuo 3635(TAIF)	Mindano, the Philippines	151,255	45.0%	OP081124
Oenotrichia maxima (E. Fourn.) Copel.	LRP 8303(WELT)	New Caledonia	148,759	42.2%	OP081113
Paesia elmeri Copel.	Kuo 2668(TAIF)	Mindano, the Philippines	150,711	43.0%	OP081115
Paesia elmeri Copel.	Kuo 3576(TAIF)	Mindano, the Philippines	150,671	43.0%	OP081104
Paesia elmeri Copel.	Kuo 3637(TAIF)	Mindano, the Philippines	150,835	43.0%	OP081134
Paesia glandulosa (Sw.) Kuhn	Fuentes A et al. 10847(MO)	La Paz, Bolivia	150,564	43.0%	OP081139
Pteridium caudatum (L.) Maxon	Jaime Canek Ledesma Corral 1816(MO)	Estado De Mexico, Mexico	152,693	41.5%	OP081096
