# Supplementary figures and images for: Plastome phylogenomic analysis reveals evolutionary divergences of Polypodiales suborder Dennstaedtiineae
Source: BMC Plant Biol. 2022 Nov 2;22:511. doi: 10.1186/s12870-022-03886-1 (PMC9628275; doi:10.1186/s12870-022-03886-1)

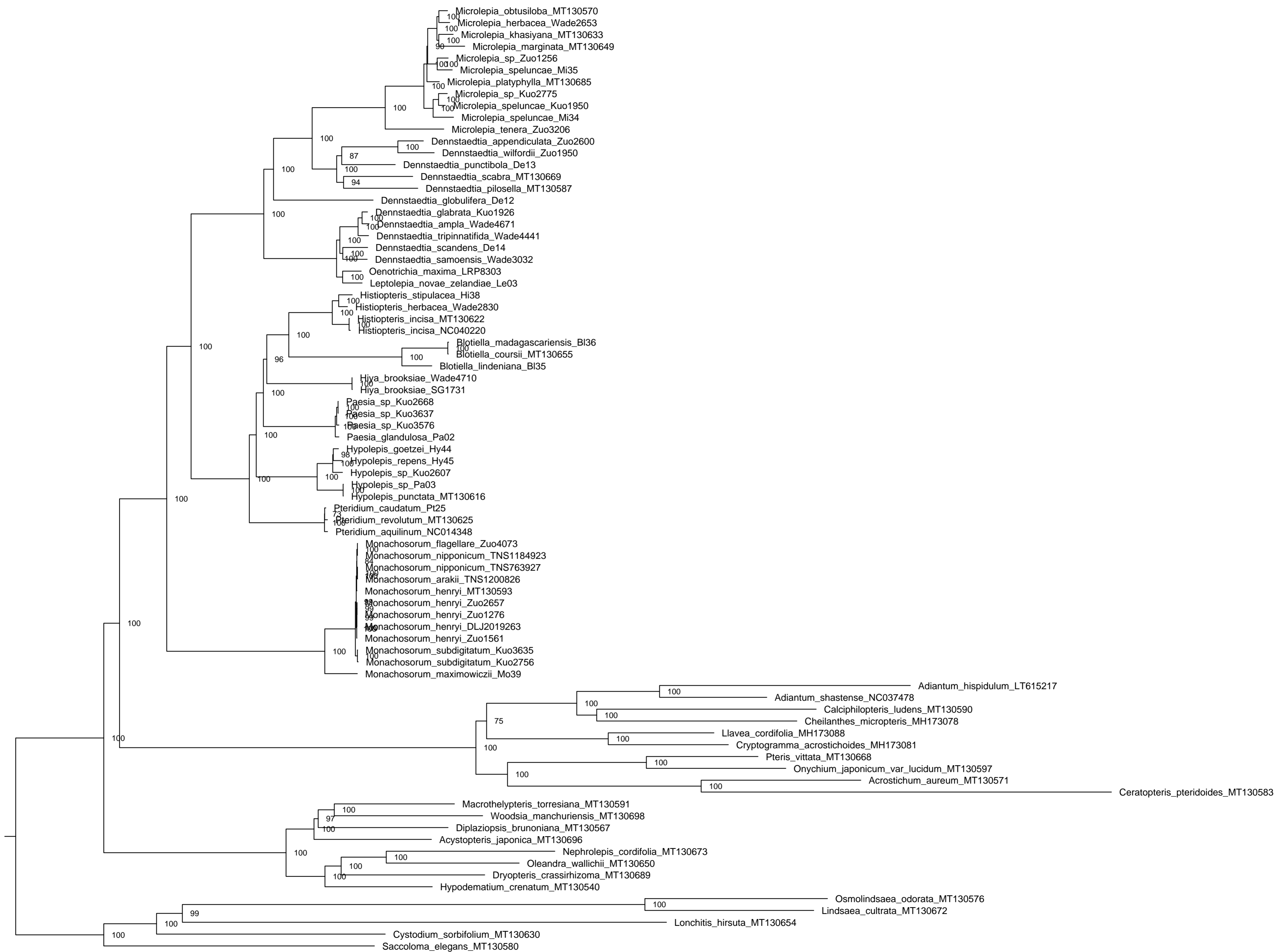

0.05

Supplement: Supplementary file 2 — Additional file 2. Maximum likelihood (ML) phylogeny of Polypodiales suborder Dennstaedtiineae based on CDS data set. [file 12870_2022_3886_MOESM2_ESM.pdf]

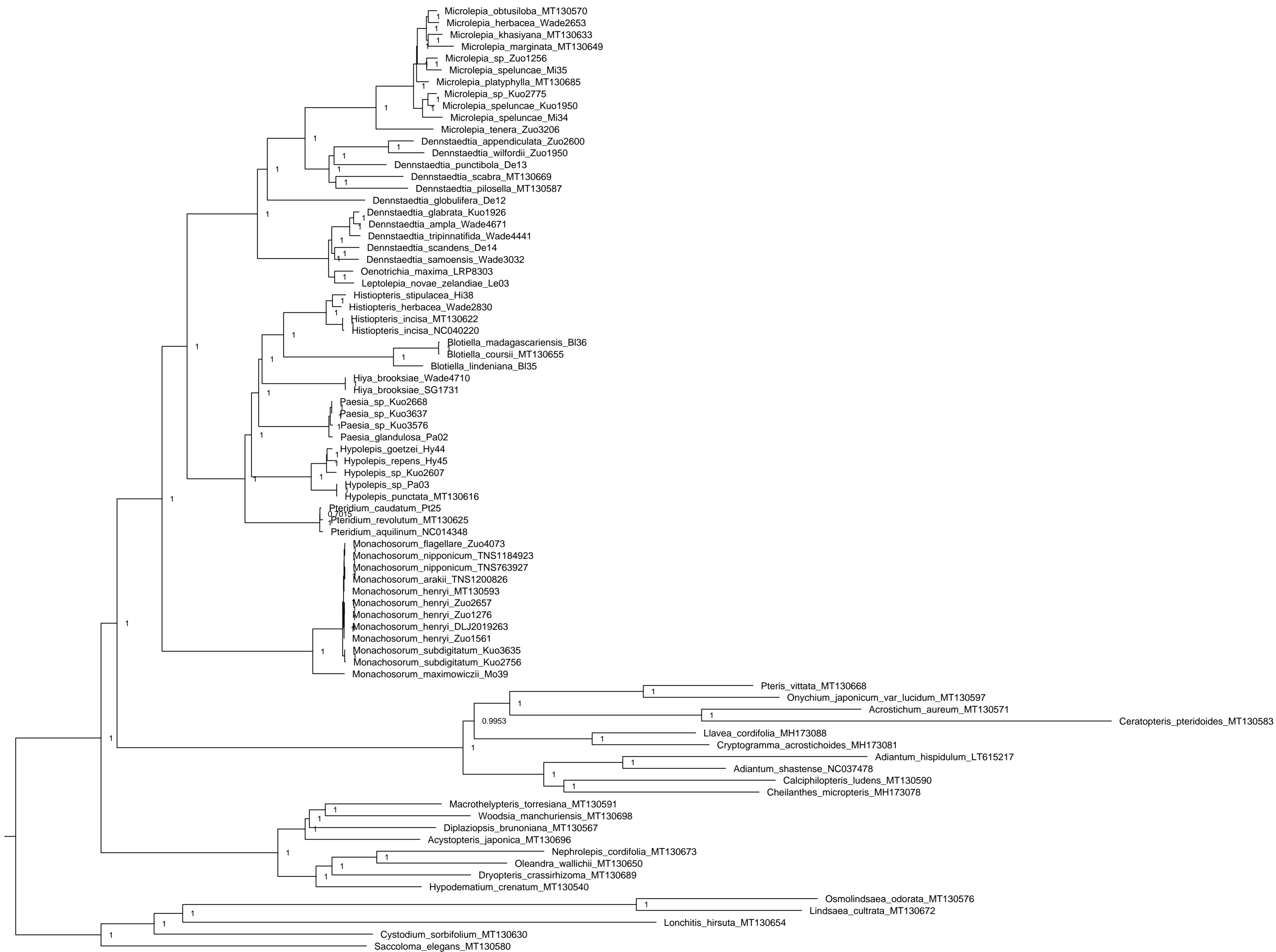

0.05

Supplement: Supplementary file 3 — Additional file 3. Bayesian inference (BI) phylogeny of Polypodiales suborder Dennstaedtiineae based on CDS data set. [file 12870_2022_3886_MOESM3_ESM.pdf]
